# Supplementary material for: rs66651343 and rs12909095 confer lung cancer risk by regulating CCNDBP1 expression
Source: PLoS One. 2023 Apr 14;18(4):e0284347. doi: 10.1371/journal.pone.0284347 (PMC10104294; doi:10.1371/journal.pone.0284347)
Supplement: S1 Table — (DOCX) [file pone.0284347.s001.docx]

Table S1. Primers in plasmid construction.

| SNP | Primer sequence | Anneal Temperature (℃) |
| --- | --- | --- |
| rs748404 and rs12911132 | CAGTCAGATCTGGTAGCTGCCTCCGGTTCCT | 60 |
|  | CAGTCCCCGGGTTGCAGAGATGATACCCCAAAAC |  |
| rs35535692 | CAGTCACGCGTCTTGGGGCTTACTGATCATTCTC | 60 |
|  | CAGTCGGTACCGTATGAGCCTTCTGAACCATTC |  |
| rs66651343 and rs12909095 | CAGTCACGCGTCTTTTGCAAAATCACCCTGTACG | 60 |
|  | CAGTCGGTACCTCTTCCTCCATTTCAGAAGGTCT |  |
| rs17779494 | CAGTCACGCGTCCTCCTCCATAACACCTACCTAG | 60 |
|  | CAGTCGGTACCATAAGCTCTTCAAAGGCAGGAAC |  |
