# Supplementary material for: rs66651343 and rs12909095 confer lung cancer risk by regulating CCNDBP1 expression
Source: PLoS One. 2023 Apr 14;18(4):e0284347. doi: 10.1371/journal.pone.0284347 (PMC10104294; doi:10.1371/journal.pone.0284347)
Supplement: S2 Table — (DOCX) [file pone.0284347.s002.docx]

Table S2. Primers in mutagenesis.

| SNP | Primer sequence^a^ | Anneal Temperature (℃) |
| --- | --- | --- |
| rs748404 | TTTGCTCACTcGCCCTCAGGT  CCAGTTGTGATCTGCTCG | 63 |
| rs12911132 | GAATCCACCAaCTATTGACTG  TCAGGTAAAGTAGCCAGC | 60 |
| rs35535692 | GAAGGGAAACaTGAGTCAGGG CATGTACACATCTTCATTGAG | 59 |
| rs66651343 | CTGTCCCCTAcTGATGGGCAT  GATTAGAAATTACTTGCACTGATACTATGAG | 65 |
| rs12909095 | ATCATCATATcGTATGTATATTTCTACAAC  ACCATGGATATACCAAAAAAG | 58 |
| rs17779494 | CTGGATAGAGaAGTTTGTGGTC  ATGACAGTTTTAACAGTCTG | 58 |

^a^The taget site in lower case.
