# Supplementary material for: rs66651343 and rs12909095 confer lung cancer risk by regulating CCNDBP1 expression
Source: PLoS One. 2023 Apr 14;18(4):e0284347. doi: 10.1371/journal.pone.0284347 (PMC10104294; doi:10.1371/journal.pone.0284347)
Supplement: S3 Table — (DOCX) [file pone.0284347.s003.docx]

Table S3. Primers in 3C-qPCR.

| Primer name | Primer sequence | Location in genome^a^ | Restrictive segment location | Target element |
| --- | --- | --- | --- | --- |
| Target primer 1 | ATCCCAGTAGTCTTTGTTCGTTTTCTTA | Chr15:43451201-43451228 | Chr15:43449694-43451254 |  |
| Target primer 2 | TTAAGGGTGCTGAGAAGACTGCTAC | Chr15:43471103-43471127 | Chr15: 43464155-43471159 |  |
| Target primer 3 | TTATTGGGGTTCCTTGCCTCAG | Chr15:43482378-43482399 | Chr15: 43473639-43482463 | *CCNDBP1* promoter |
| Target primer 4 | TACTCCCCAGCCTCACCTTTTC | Chr15:43496296-43496317 | Chr15: 43490452-43496388 |  |
| Target primer 5 | AGAGGAGCAAAGACAGGGAAGTG | Chr15:43506680-43506702 | Chr15: 43503976-43506729 |  |
| Target primer 6 | ACATAAAGGTGAAAAAGAATGAAGTAGCTC | Chr15:43516904-43516933 | Chr15: 43509558-43516962 | *EPB42* promoter |
| Target primer 7 | GGTCGAAGGGAAGGTGTATGC | Chr15:43527879-43527899 | Chr15: 43525351-43527976 |  |
| Target primer 8 | CTTTACTGCCACACTCCCATAGC | Chr15:43537422-43537444 | Chr15: 43535603-43537548 |  |
| Target primer 9 | ATGTTTTTCCCACTATCCTTCACTTG | Chr15:43546949-43546974 | Chr15: 43544399-43547008 |  |
| Target primer 10 | GGTGAGACCAATAGCGATACTTTTTC | Chr15:43560122-43560147 | Chr15: 43558457-43560171 | *TGM5* promoter |
| Target primer 11 | AAGAGGTGATTCGATGGTGATGG | Chr15: 43573939-43573961 | Chr15: 43560351-43574003 |  |
| Target primer 12 | TCTAGTGGTGGAGTGGGGAGTG | Chr15: 43593057-43593082 | Chr15: 43591345-43593118 |  |
| Target primer 13 | CCAAGGGGAAAAGATATGCTAAACTC | Chr15: 43596706-43596730 | Chr15: 43595971-43596776 | *TGM7* promoter |
| Target primer 14 | ATACTGATGCGGTGGAAGAAAAATA | Chr15: 43605163-43605187 | Chr15: 43598650-43605212 |  |
| Constant primer 1 | ACACAAAAAGTAATAAATAAAGGTAAGGTTGTT | Chr15: 43558403-43558435 | Chr15:43557561-43558457 | enhancer containing  rs66651343 and rs12909095 |
| Constant primer 2 | TCACTGAAGAGCCCCCAAGAA | Chr15: 43578295-43578315 | Chr15:43574003-43578422 | enhancer containing rs17779494 |

^a^Relative to human genome build37.
