# Supplementary material for: rs66651343 and rs12909095 confer lung cancer risk by regulating CCNDBP1 expression
Source: PLoS One. 2023 Apr 14;18(4):e0284347. doi: 10.1371/journal.pone.0284347 (PMC10104294; doi:10.1371/journal.pone.0284347)
Supplement: S4 Table — (DOCX) [file pone.0284347.s004.docx]

Table S4. Primers in ChIP-qPCR.

| SNP | Primer sequence |
| --- | --- |
| rs66651343 | GCACACAGAGGCATGGATAA  ATGTCTGGGAGCTCTGTCCA |
| rs12909095 | GGTACTGACTGGCATGGACA  GGATTAGCATGTGTGGTCTTTTT |
