# Supplementary material for: rs66651343 and rs12909095 confer lung cancer risk by regulating CCNDBP1 expression
Source: PLoS One. 2023 Apr 14;18(4):e0284347. doi: 10.1371/journal.pone.0284347 (PMC10104294; doi:10.1371/journal.pone.0284347)
Supplement: S5 Table — (DOCX) [file pone.0284347.s005.docx]

Table S5. Probes for rs66651343 and rs12909095 in EMSA.

| SNP | Allele | Probe sequence^a^ |
| --- | --- | --- |
| rs66651343 | A allele  G allele | TAAATGCCCATCAATAGGGGACAGGAT ATCCTGTCCCCTATTGATGGGCATTTA  TAAATGCCCATCAGTAGGGGACAGGAT ATCCTGTCCCCTACTGATGGGCATTTA |
| rs12909095 | A allele  G allele | GAAATATACATACAATATGATGATACC  GGTATCATCATATTGTATGTATATTTC  GAAATATACATACGATATGATGATACC  GGTATCATCATATCGTATGTATATTTC |

^a^The target sites underlined.
