# Supplementary material for: rs66651343 and rs12909095 confer lung cancer risk by regulating CCNDBP1 expression
Source: PLoS One. 2023 Apr 14;18(4):e0284347. doi: 10.1371/journal.pone.0284347 (PMC10104294; doi:10.1371/journal.pone.0284347)
Supplement: S6 Table — (DOCX) [file pone.0284347.s006.docx]

Table S6. *r^2^* value between rs748404 and other SNPs in three representative populations.

|  | CEU | CHB | YRI |
| --- | --- | --- | --- |
| rs35535692^a^ | 0.841 | - | - |
| rs66651343 | 1.000 | 1.000 | 0.426 |
| rs12909095 | 1.000 | 0.336 | 0.426 |
| rs12911132 | 1.000 | 1.000 | 0.426 |
| rs17779494 | 1.000 | 1.000 | 0.426 |

^a^Not in polymorphism in CHB and YRI.
