# Supplementary material for: rs66651343 and rs12909095 confer lung cancer risk by regulating CCNDBP1 expression
Source: PLoS One. 2023 Apr 14;18(4):e0284347. doi: 10.1371/journal.pone.0284347 (PMC10104294; doi:10.1371/journal.pone.0284347)
Supplement: S7 Table — (DOCX) [file pone.0284347.s007.docx]

Table S7. Primers in RT-PCR.

| Gene | Primer sequence |
| --- | --- |
| *EPB42* | ACCCAAGTGCTCCTAATGGAGG CCATCCTCACAGCACTTCCAGA |
| *TGM5* | GCACAAAGAGCATCCAGAGTGAC AGAGCCTTCAGAAACACCTGCC |
| *TGM7* | CACAACACCAGTTCCATCGGGA  CCAGCATTTTCCGAGAAGCCTTC |
| *GAPDH* | GAAGGTGAAGGTCGGAGTC  GAAGATGGTGATGGGATTTC |
