# Supplementary figures and images for: rs66651343 and rs12909095 confer lung cancer risk by regulating CCNDBP1 expression
Source: PLoS One. 2023 Apr 14;18(4):e0284347. doi: 10.1371/journal.pone.0284347 (PMC10104294; doi:10.1371/journal.pone.0284347)

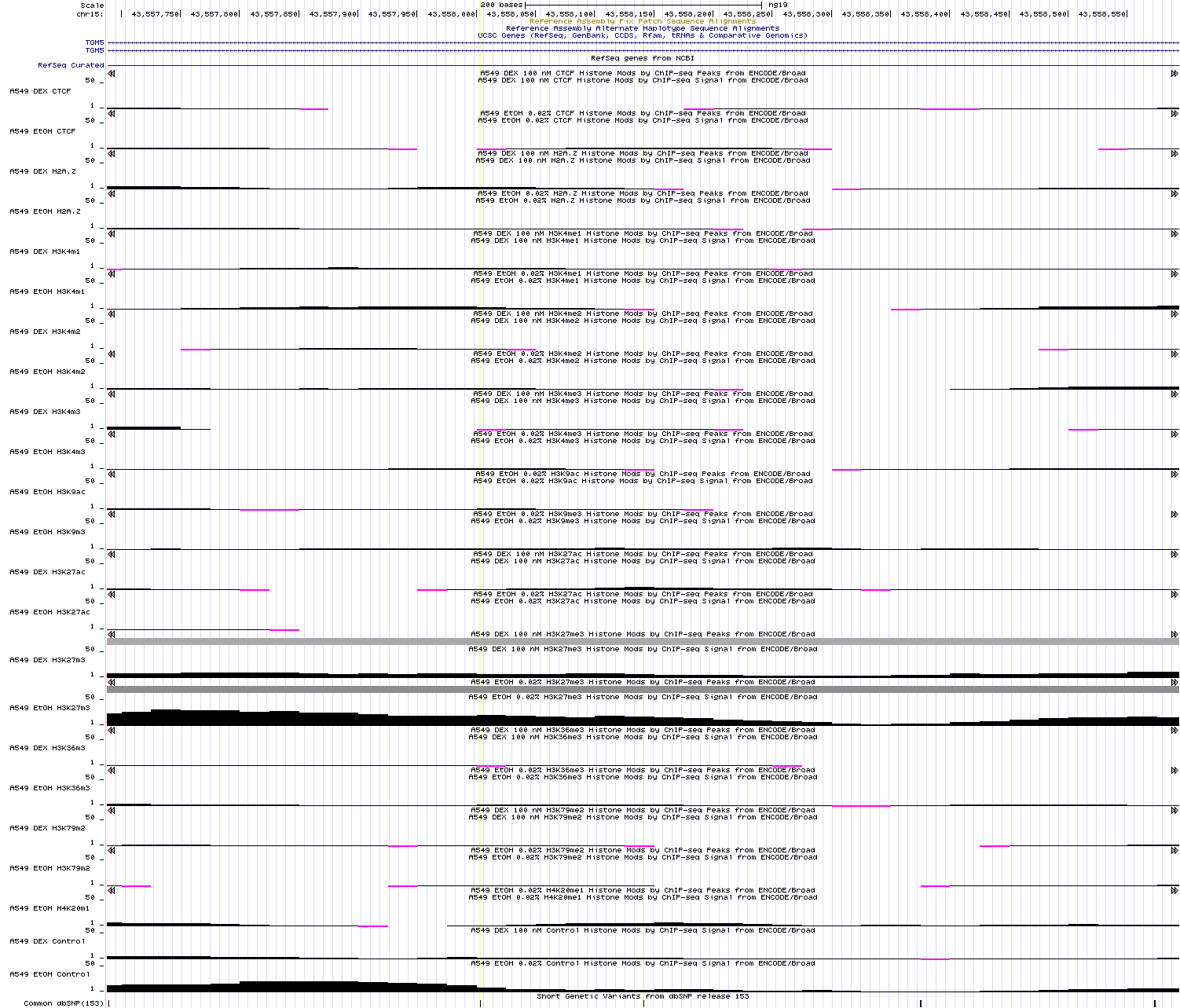

Supplement: S1 Fig — The yellow lines in left and right indicate the location of rs66651343 and rs12909095, respectively. (TIF) [file pone.0284347.s008.tif]

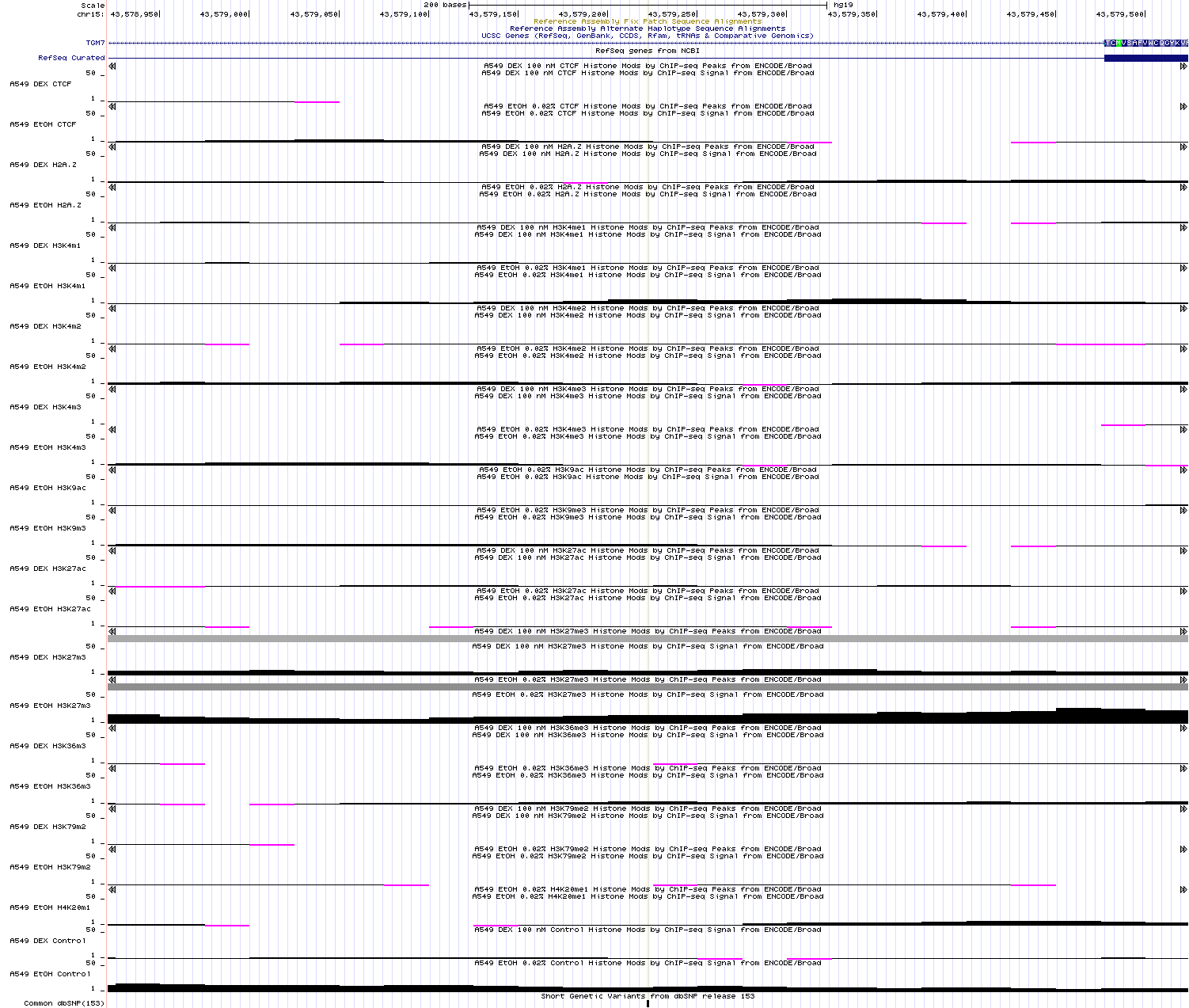

Supplement: S2 Fig — The yellow line indicates the location of rs17779494. (TIF) [file pone.0284347.s009.tif]
