# Supplementary material for: rs66651343 and rs12909095 confer lung cancer risk by regulating CCNDBP1 expression
Source: PLoS One. 2023 Apr 14;18(4):e0284347. doi: 10.1371/journal.pone.0284347 (PMC10104294; doi:10.1371/journal.pone.0284347)

Original RT-PCR result. The lanes 1-3, 4-6 and 7-9 are for genes *EPB42*, *TGM5* and *TGM7*, respectively. The lanes 1, 4 and 7 are molecular weight marker. Fig 3 is generated from this gel image.

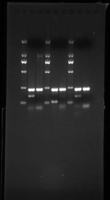

Original EMSA result for rs66651343 in Beas-2B cell. The lanes 1-3 are for allele A while 4-6 for G. The Fig 7A is generated from this gel image.

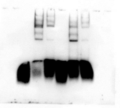

Original EMSA result for rs12909095 in Beas-2B cell. The lanes 1-3 are for allele A while 4-6 for G. The Fig 7A is generated from this gel image.

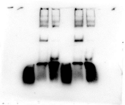

Original EMSA result for rs66651343 in A549 cell. The lanes 1-3 are for allele A while 4-6 for G. The Fig 7B is generated from this gel image.

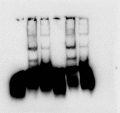

Original EMSA result for rs12909095 in A549 cell. The lanes 1-3 are for allele A while 4-6 for G. The Fig 7B is generated from this gel image.

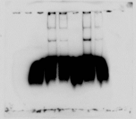

Supplement: S1 Raw images — (PDF) [file pone.0284347.s010.pdf]
