# Supplementary material for: rs66651343 and rs12909095 confer lung cancer risk by regulating CCNDBP1 expression
Source: PLoS One. 2023 Apr 14;18(4):e0284347. doi: 10.1371/journal.pone.0284347 (PMC10104294; doi:10.1371/journal.pone.0284347)
Supplement: Attachment — Submitted filename: Response to Reviewers.docx [file pone.0284347.s011.docx]

Dear Dr. Yan-Ming Xu,

Thank you for giving us the opportunity to revise our manuscript entitled “rs66651343 and rs12909095 confer lung cancer risk by regulating *CCNDBP1* expression”. We have revised our manuscript according to the comments of the two reviewers and described our responses and the changes to the manuscript in detail in the attached sheet. All revisions have been marked as red.

Our original gel image data are provided in Supporting Information.

We hope you will find the manuscript acceptable for publication in *PLoS ONE.*

Thank you for your consideration.

Sincerely,

Chang Sun

**Reviewer 1:**
*The Beas-2B cells cultured with 10% FBS medium will transform, so the results are not reliable;*

We verified our conclusions in another lung cancer cell line A549 and included the results.

*The figures are intricate with the simple labels and legends;*

We revised the figure lables and legends.

*Could you show the mRNA or protein level of the gene regulated by the SNPs?*

We talked about this issue in Discussion.

**Reviewer 2:**

*The authors used normal human lung BEAS-2B cells for many of their experiments, but FBS was used to culture the cells and this would have caused the transformation of the cell line – which may impact the results obtained. Therefore, all these results should be retested, and if possible, in multiple cell lines.*

We verified our conclusions in another lung cancer cell line A549 and included the results.

*Unfriendly mode of English can be found.*

We revised the text thoroughly.

*Figures have to be more “compact”, e.g. the authors can merge a few of the main figures into 1 figure.*

We merged the previous luciferase, 3C and eQTL results into 1 figure.

*Many of the supplementary figures could become main figures (e.g., Figures S1, S4–S7).*

We put the previous Fig S1 and S4-7 into main figures.

*The authors claimed that rs748404, a relatively well-characterized lung cancer risk-associated SNP, is not functional. This statement requires further verification and/or discussion (in both Results and Discussion sections).*

We verified the conclusion in another cell line A549 and talked about this issue in Discussion.
