# Supplementary material for: rs66651343 and rs12909095 confer lung cancer risk by regulating CCNDBP1 expression
Source: PLoS One. 2023 Apr 14;18(4):e0284347. doi: 10.1371/journal.pone.0284347 (PMC10104294; doi:10.1371/journal.pone.0284347)
Supplement: Attachment — Submitted filename: Response to Reviewers.docx [file pone.0284347.s012.docx]

Dear Dr. Yan-Ming Xu,

Thank you for giving us the opportunity to revise our manuscript entitled “rs66651343 and rs12909095 confer lung cancer risk by regulating *CCNDBP1* expression”. We have revised our manuscript according to the comments of the reviewer and described our responses and the changes to the manuscript in detail in the attached sheet. All revisions have been marked as red.

We hope you will find the manuscript acceptable for publication in *PLoS ONE.*

Thank you for your consideration.

Sincerely,

Chang Sun

**Reviewer 2:**
1. *Data regarding A549 cells can be included in the main figures.*

We have included A549 cell results in main figures.

2. *Similar results obtained with the A549 cells did not change the fact that FBS would transform BEAS-2B cells – this should be mentioned or discussed in the manuscript.*

We added one paragraph in Discussion to talk about this issue.

3. *It was claimed that rs748404 was not a “functional” SNP, but why the short title of the manuscript stated: “association between rs748404 and lung cancer”?*

We revised the short title as “Function of 15q15.2 SNPs in lung cancer”.

4. *English writing of the manuscript can be further polished.*

We invited a native speaker to revise the text and improve the language.
