# Supplementary material for: rs66651343 and rs12909095 confer lung cancer risk by regulating CCNDBP1 expression
Source: PLoS One. 2023 Apr 14;18(4):e0284347. doi: 10.1371/journal.pone.0284347 (PMC10104294; doi:10.1371/journal.pone.0284347)
Supplement: Attachment — Submitted filename: Response to Reviewers.docx [file pone.0284347.s013.docx]

Dear Dr. Yan-Ming Xu,

Thank you for giving us the opportunity to revise our manuscript entitled “rs66651343 and rs12909095 confer lung cancer risk by regulating *CCNDBP1* expression”. We have revised our manuscript according to the comments of the Editor and described our responses and the changes to the manuscript in detail in the attached sheet. All revisions have been marked as red.

We hope you will find the manuscript acceptable for publication in *PLoS ONE.*

Thank you for your consideration.

Sincerely,

Chang Sun

**Editor:**
*"Our study has one limitation in that FBS, although widely used in tissue culture, might transform Beas-2B cell, which might further bias the result. To validate our conclusions, we investigated the function and effect of related SNPs in another lung cancer cell line A549. The similar patterns between these two cell lines indicate that the potential transform should not alter the trans-regulatory environment in cell and influence the function of related SNPs."*

*Should be corrected as*

*"Our study has one limitation in that FBS, although widely used in tissue culture, might transform Beas-2B cell, which might further bias the result. To validate our conclusions, we investigated the function and effect of related SNPs in another lung cell line A549. The similar patterns between these two cell lines indicate that the potential Beas-2B cell transformation should not alter the trans-regulatory environment in cell and influence the function of related SNPs."*

We revised related text.

*Before it can be formally accepted, the authors are required to perform thorough English grammar check and polishing for all the content throughout the article files.*

We revised the grammar and polished the text.
